# Self‐Oscillation in Active Wires with Asymmetric Willis‐Type Viscosity

**DOI:** 10.1002/advs.202500737

**Published:** 2025-04-17

**Authors:** Xingbo Pu, Xiaoyu Hou, Antonio Palermo, Yangyang Chen

**Affiliations:** ^1^ Department of Mechanical and Aerospace Engineering The Hong Kong University of Science and Technology Kowloon, Clear Water Bay 999077 Hong Kong; ^2^ Department of Civil, Chemical Environmental and Materials Engineering University of Bologna Bologna 40136 Italy

**Keywords:** active metamaterial, self‐oscillation, Willis material

## Abstract

Wires designed to carry loads through uniaxial tensile forces are particularly useful in creating large‐span, yet lightweight, structures of diverse shapes and functions. Incorporating self‐oscillation capabilities within the wires could be a promising approach to enhance the autonomous dynamic functions of these structures. However, self‐oscillation, which usually appears in biological organisms and active materials, requires complex feedback interactions between activity and elasticity, hindering their application in self‐oscillating wires. Here, a simple self‐oscillation strategy is suggested by extending Willis elasticity to *Willis‐type viscosity* through an irreversible coupling between strain rate and body force. A class of *active wires* equipped with electro‐magneto‐mechanically coupled feedforward loops is designed to realize the irreversible coupling. Numerical experiments show that the active wire hosts biased limit‐cycle self‐oscillation, where oscillation amplitudes are amplified linearly along one direction. Using continuum models, the linear amplification is interpreted as the force equilibrium caused by asymmetric Willis viscosity. In the design, the oscillation mode shape can be tailored independently of frequency, and the active wire supports the standing propagating mode transition. The design and continuum model suggested can benefit the development of autonomous materials for large‐span structures.

## Graphical abstract

This study suggests a self‐oscillation strategy enabled by a new set of material parameters, namely the asymmetric Willis‐type viscosity, to bias and independently control oscillation mode shapes as well as frequencies. The exemplified active wire can benefit the development of autonomous materials to construct soft motors, agitators, pumps, and shakers.

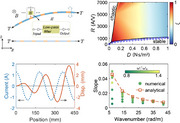

## 1 Introduction

Wires, cables, and ropes are basic mechanical components that exhibit rigidity when subjected to uniaxial tension, but are highly flexible when exposed to other types of loads. This distinctive property makes them ideal for the construction of lightweight planar and shell structures, i.e., large‐span architectures, Ferris wheels, and cableways.^[^ ^1^, ^2^, ^3^ ^]^ Fine control of axial tension can tune the response of wires and, in turn, the mechanical behavior of lattice‐type materials that incorporate cable systems, such as tensegrity structures or spiderweb‐like lattices.^[^ ^4^, ^5^, ^6^, ^7^ ^]^ Nonetheless, passive control strategies based on prestress tuning cannot introduce complex functionalities, such as sensing or autonomous tuning.

On the other hand, active materials incorporated with microscopic engines offer unparalleled opportunities to design structural systems with autonomous functions.^[^ ^8^, ^9^, ^10^, ^11^, ^12^ ^]^ Among them, self‐oscillation energized by a source of power without periodicity is a unique but important form of dynamic functions, particularly useful for self‐propulsion, self‐organization, and autonomous tuning.^[^ ^13^, ^14^, ^15^, ^16^ ^]^ Recently, the ability of self‐oscillation has been introduced to active metamaterials, i.e., mechanical lattices with sensing, actuating, and computation components.^[^ ^17^, ^18^, ^19^, ^20^, ^21^, ^22^ ^]^ Yet, research effort toward active wires has never been advanced.

Here, our objective is to extend autonomous self‐oscillation capabilities to mechanical wires to enhance the dynamic functions of their embedding structures and to promote new applications in locomotion and swimming of wide‐span robots, as well as in planar and shell agitators to enhance material synthesis, and to improve the performance of thermal and fluid devices.^[^ ^23^, ^24^, ^25^, ^26^, ^27^, ^28^, ^29^, ^30^, ^31^, ^32^, ^33^ ^]^ In general, self‐oscillation arises due to instabilities of linearized equations of motion, so that perturbations grow exponentially with time. Often, the instability is induced by positive feedback loops of microscopic engines, and the systems are therefore characterized by negative viscosity or negative damping, i.e., producing a feedback force based on the velocity that pushes a particle forward when the particle moves forward.^[^ ^34^, ^35^, ^36^ ^]^ When the nonlinear elasticity of the systems becomes significant, the exponential growth of the oscillation amplitudes is blocked, giving rise to limit‐cycle self‐oscillations.^[^ ^37^, ^38^ ^]^ Self‐oscillation is thus the result of complex feedback interactions between activity and elasticity. However, the distinctive mechanical properties of wires restrict the direct application of this mechanism to design self‐oscillating wires.

**Figure 1:**
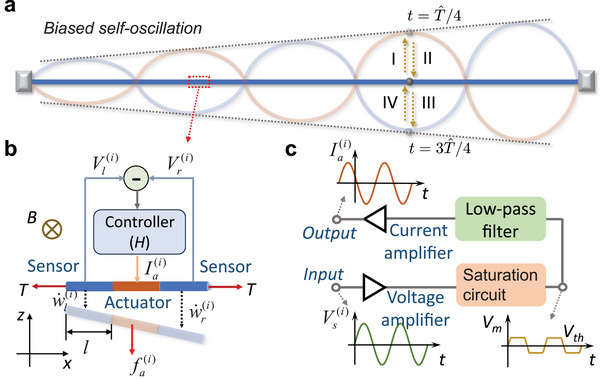
Microstructural design of an active wire hosting biased self‐oscillation. a) The displacement field profiles at two time steps $t=\hat{T}/4$ and $t=3\hat{T}/4$, where $\hat{T}$ is the period of self‐oscillation. The oscillation amplitude is amplified linearly along one direction. The blocks positioned at the ends of the wire serve as a visual representation of the fixed‐end boundary condition. b) The active wire consists of three segments of metal wire placed in a magnetic field and connected to an electrical controller. c) The electrical controller contains a voltage amplifier, a nonlinear saturation circuit, a low‐pass filter, and a current amplifier.

In this study, we suggest another self‐oscillation strategy enabled by a new set of response coefficients, called *Willis‐type viscosity*, that creates the coupling between the strain rate and body force. We design a class of *active wires* that utilize feedforward electro‐magneto‐mechanical coupled loops for sensing, computing, and actuating to realize an asymmetric form of Willis‐type viscosity in a one‐dimensional space. The active wire self‐oscillates in a limit cycle but with biased mode shapes, that is, the oscillation amplitude is linearly amplified along one direction (see **Figure** **1**a). Additionally, the biased mode shapes can be tailored independent of frequencies. We interpret the biased limit‐cycle self‐oscillation and quantify the linear amplification using a continuum model built upon Willis‐type viscosity. Finally, the standing‐to‐propagating mode transition is found by tuning the passive damping of the active wire. The physical principle discovered in this study can be exploited to understand nonstandard self‐oscillations in other man‐made materials, and the active wire design can promote the development of autonomous materials used for materials synthesis and fabrication, as well as fluid‐structure interactions.

## 2 Results

### 2.1 Design of The Active Wire

The microstructure of the active wire consists of three segments of straight metal wire with length *l* and tension force *T* placed in a magnetic field *B* and connected to an electric circuit (see Figure 1b). The mass per unit length, damping, and viscosity coefficients of the metal wire are denoted by ρ, *D*, and η, respectively. The metal wire moves along *z*‐direction with displacement *w*, perpendicular to the magnetic field *B*. The two wire segments on the left and right sides of the *i*‐th unit cell (blue lines) function as sensors that convert mean velocities ${\dot{w}}_{l}^{\left(i\right)}$ and ${\dot{w}}_{r}^{\left(i\right)}$ of the two sensing wires to two electrical voltages $V_{l}^{\left(i\right)}={\dot{w}}_{l}^{\left(i\right)}Bl$ and $V_{r}^{\left(i\right)}={\dot{w}}_{r}^{\left(i\right)}Bl$, respectively, according to Faraday's law. We take the difference between the two voltages $V_{s}^{\left(i\right)}=V_{l}^{\left(i\right)}-V_{r}^{\left(i\right)}$ of the *i*‐th unit cell as the input signal of its electric circuit such that its mean strain rate, ${\dot{s}}_{m}^{\left(i\right)}=\frac{{\dot{w}}_{l}^{\left(i\right)}-{\dot{w}}_{r}^{\left(i\right)}}{2l}=\frac{V_{s}^{\left(i\right)}}{2Bl^{2}}$ is measured. The wire segment in the middle of the unit cell (orange line) serves as an actuator. When an actuating current $I_{a}^{\left(i\right)}$ flows through the wire, a magnetic body force $f_{a}^{\left(i\right)}=I_{a}^{\left(i\right)}B$ is generated on the wire. An electric circuit is implemented between the sensors and the actuator to create a new mechanical response between the body force and the strain rate on the active wire. In particular, the electric circuit comprises a voltage amplifier, a nonlinear saturation circuit, a second‐order low‐pass filter, and a current amplifier (see Figure 1c). Voltage and current amplifiers in the circuit modulate the ratio between the magnitudes of body force and strain rate to induce linear instability. The nonlinear saturation circuit enforces a threshold voltage *V*_*th*_ to suppress the exponential growth of the oscillation amplitude once self‐oscillation occurs. The low‐pass filter in the circuit manipulates the phase delay between body force and strain rate, a parameter also controlling the linear stability of the active wire. Finally, it may need to mention that Joule heating is inevitable in the active wire, similar to what occurs in motors and generators. In practical applications, Joule heating should be properly managed to increase efficiency and, in the meantime, to avoid overheating (see detailed discussion in Supporting Information).

### 2.2 Self‐Oscillation of The Active Wire

**Figure 2:**
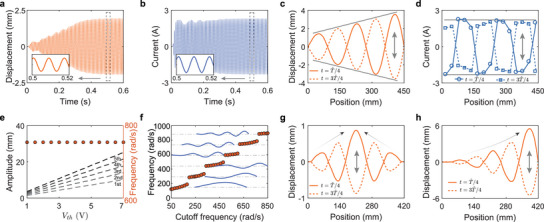
Numerical simulations of the biased self‐oscillation. a,b) Displacement (a) and actuating current (b) evolved in time at the central point of the unit cell near the right boundary. c,d) Displacement fields (c) and current profiles (d) on the entire wire at two‐time steps when they reach maximum and/or minimum values. e) Self‐oscillation frequencies and amplitudes at five peaks and/or valleys of the active wire with different *V*_*th*_. f) Self‐oscillation frequencies and the corresponding mode shapes of the active wire with different ω_*c*_. In the simulations (a‐e), we select copper wires thanks to the following reasons: (i) Copper wire has high electrical conductivity, allowing for efficient current transmission; (ii) Copper wire has relatively high tensile strength, able to support required pulling forces; (iii) Copper wire is easy to process, allowing for various connections. Base on this selection, the parameters ρ = 4.5 g/m, *T* = 2 N, *D* = 0.15 N · s/m^2^, η = 0, *B* = 1 T, ω_*c*_ = 700 rad/s, μ = 0.5, *l* = 10 mm, *R* = 1000 A/V, and the total length of the active wire *l*_*w*_ = 0.45 m. g,h) Displacement profiles of the active wire composed of 15 unit cells at two‐time steps when they reach maximum and/or minimum values. g) *R* = 5 × 10^3^ A/V for the left 8 unit cells and *R* = −5 × 10^3^ A/V for the right 7 unit cells. h) The threshold voltage varies linearly in space following the function $V_{th}^{\left(n+1\right)}=V_{th}^{\left(1\right)}+0.5n$.

To examine the self‐oscillation of the active wire, we perform time‐dependent numerical simulations. Equations of motion of the active wire are formulated in both mechanical and electrical domains as

(1) $$\rho \ddot{w}+D\dot{w}-T\frac{\partial^{2}w}{\partial x^{2}}-\eta \frac{\partial^{2}\dot{w}}{\partial x^{2}}-\sum_{i=1}^{N}BI_{a}^{\left(i\right)}\left[x_{l}^{\left(i\right)}\leq x\leq x_{r}^{\left(i\right)}\right]=0$$

(2) $$\frac{1}{\omega_{c}^{2}}{\ddot{I}}_{a}^{\left(i\right)}+\frac{\mu }{\omega_{c}}{\dot{I}}_{a}^{\left(i\right)}+I_{a}^{\left(i\right)}=\left\{\begin{array}{c} RV_{s}^{\left(i\right)},\left|RV_{s}^{\left(i\right)}\right|<V_{th},\\ \text{sgn}\left(V_{s}^{\left(i\right)}\right)V_{th},\;\left|RV_{s}^{\left(i\right)}\right|\geq V_{th}. \end{array}\right.$$

where [·], *N*, $x_{l}^{\left(i\right)}$, and $x_{r}^{\left(i\right)}$ denote the Iverson bracket, number of the unit cells, positions of the left and right boundaries of the *i*‐th actuating wire, respectively, and ω_*c*_, μ, and *R* are the cutoff frequency, damping coefficient, and linear amplification ratio of the circuit, respectively. **Figure** **2**a,b show the solutions of *w* and $I_{a}^{\left(i\right)}$ as a function of time in the center of the unit cell near the right boundary (*i* = *N* = 15) (see Supporting Information for simulation details). It can be seen that both displacement *w* and actuating current $I_{a}^{\left(i\right)}$ experience fast exponential growth from their initial position during the first three cycles. After that, the nonlinear circuit becomes saturated, and the exponential growth of displacement turns into a quasi‐linear growth until 0.4 s. Then, both the displacement *w* and the actuating current $I_{a}^{\left(i\right)}$ oscillate stably with constant amplitudes and at the same frequency *f*_*o*_ ≈ 120 Hz, which is close to the cutoff frequency of the circuit $\frac{\omega_{c}}{2\pi }$. Clearly, the self‐oscillation of the active wire is a limit‐cycle oscillation. Note that different initial conditions would affect the time evolution of the self‐oscillation of the active wire before it reaches the limit‐cycle oscillation. However, the limit‐cycle oscillation remains invariant when the initial conditions change (see Supporting Information for details). Further, comparing the inset figures in Figure 2a,b, it can be found that the displacement *w* is almost in phase with the actuating current $I_{a}^{\left(i\right)}$, implying that the body force generated by the actuating current has a π/2 phase difference with the velocity. As a result, the average power produced by the actuating current is close to zero, and the self‐oscillation is not driven by the work done by the actuating current on the moving wire. This simple and direct driving strategy, which we call the primary drive in the study, leads to a negative damping on the system. Notably, negative damping is common in other self‐oscillating systems, but is not the main driver in the active wire.

Besides the evolution of displacement and actuating current in time, it is interesting to examine their profiles in space. Figure 2c,d shows the spatial profiles of the displacement and actuation current of the active wire when their maximum and minimum values in time are reached. Standing waves on the active wire are evident from the figures. Remarkably, the displacement of the active wire is spatially biased, exhibiting linear amplification of oscillation amplitudes along one direction. Conversely, the spatial profiles of the actuating current are close to square waves without unidirectional amplification, a consequence of the saturation circuit implemented in the design. It can also be found that the spatial phase difference between the actuating current and the displacement is around π/2. As a result, the actuating current and the displacement are in phase on some points of the active wire (e.g., the one shown in Figure 2a,b), but out of phase on other points of the active wire. This spatially shifted square wave breaks the symmetry of the oscillation mode shape and is responsible for the linear amplification. We call the driving strategy induced by the spatially shifted actuating current the secondary drive. This behavior is in stark contrast with the oscillations of conventional wires, where the amplitudes on the peaks and valleys are the same along uniform wires.^[^ ^39^ ^]^ Although unidirectional amplification has been found in active materials that display non‐Hermitian skin effects, the amplification of those materials is exponential, not linear, and without the presence of self‐oscillation.^[^ ^20^, ^40^ ^]^ It should also be noticed that the rotational motion usually observed in passive wires would not occur in the active wire, as the electromagnetic force is always aligned in the vertical plane so that the motion in the horizontal plane as well as the rotational modes are effectively eliminated (see Supporting Information for details).

In addition to biased self‐oscillation, the active wire also exhibits distinctive features in the control of mode shapes and oscillation frequencies. We first examine the effects of the saturation voltage *V*_*th*_. As shown in Figure 2e, the oscillation amplitudes in the five peaks of the active wire increase linearly with the saturation voltage, where the five peaks denote the spatial local maxima in the oscillation amplitude profile along the length of the active wire shown in Figure 2c. Furthermore, the spatial slope of this linear amplification is also proportional to the saturation voltage. Noticeably, the self‐oscillation frequency remains the same when the saturation voltage varies (see Figure 2e). Next, we examine the effects of the cutoff frequency of the electrical circuit ω_*c*_. As shown in Figure 2f, the self‐oscillation frequency shifts following the increase of the cutoff frequency of the electrical circuit and jumps between each of the modes. The oscillation amplitude also varies slightly as the cutoff frequency alters, while the linear amplification in space is still preserved among all modes (see Figure 2f). In addition to the cutoff frequency, the diameter of the wire also affects the self‐oscillation of the active wire, where independent control of frequency and mode shape is still observed (see Supporting Information for details).

Next, we explore the possibility of flexibly controlling oscillation mode shapes using a disordered design. In practice, inhomogeneity can be easily achieved by varying electrical circuit parameters in space, i.e., the amplification ratio and the threshold voltage. To show this, we consider an active wire composed of 15 unit cells with *B* = 1 T, *D* = 1 N · s/m^2^, ω_*c*_ = 700 rad/s and *V*_*th*_ = 1 V and enforce *R* = 5 × 10^3^ A/V for the left 8 unit cells and *R* = −5 × 10^3^ A/V for the right 7 unit cells. Consequently, linear amplification toward the left (right) is observed in the right‐hand (left‐hand) of the active wire, and the vibration is localized on the two boundaries of the design (see Figure 2g). In Figure 2h, we let *D* = 1 N · s/m^2^, ω_*c*_ = 700 rad/s and *R* = 5 × 10^3^ A/V, but impose $V_{th}^{\left(n+1\right)}=V_{th}^{\left(1\right)}+0.5n$, where *n* denotes the sequential number of the unit cell starting from the left boundary. As illustrated in the figure, a quasi‐quadratic envelope function is formed on the active wire when *V*_*th*_ is distributed linearly in space. It is worth mentioning that the oscillation frequency of the disordered design remains unchanged and the mode shape functions that can be realized on disordered active wires are not limited to the two demonstrations in the figure.

### 2.3 Asymmetric Willis‐Type Viscosity

**Figure 3:**
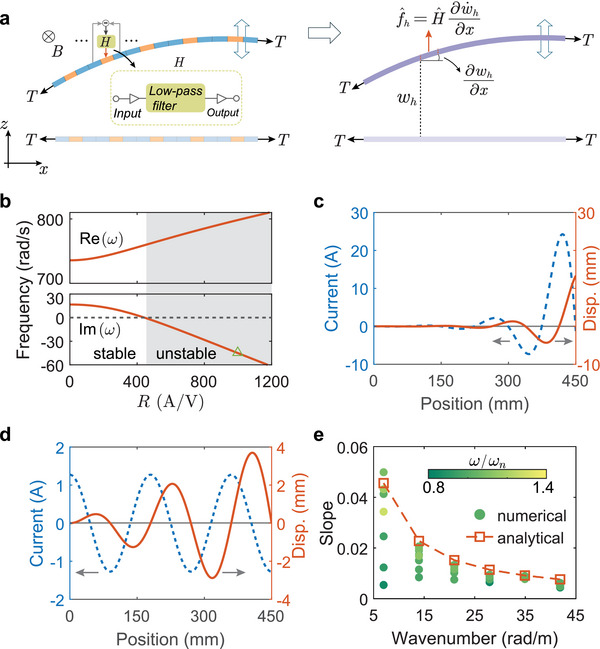
Continuum modeling of the biased self‐oscillation. a) Homogenized model. b) Eigenfrequencies of the active wire calculated from the linearized continuum model with different *R*. c) Corresponding mode shapes of the active wire attained from the linearized continuum model. d) Displacement field and actuating current profile of the active wire obtained from the nonlinear continuum model. The saturation voltage *V*_*th*_ = 1 V. e) Amplification slopes of the biased self‐oscillation with different wavenumber *k*_*n*_.

To understand the distinctive self‐oscillation behaviors of the active wire, we first homogenize the microstructure into a set of effective material parameters (see **Figure** **3**a). Clearly, the new mechanical response between body force and strain rate in the active wire cannot be described by conventional viscosity and damping coefficients. Inspired by the Willis coupling developed in the context of elastic homogenization,^[^ ^41^, ^42^ ^]^ we extend the definitions of conventional viscosity and damping coefficients to Willis‐type viscosity. In doing so, the two independent relationships between stress and strain rate as well as between body force and velocity are reorganized into two coupled constitutive equations, and the active wire can be homogenized as

(3) $$\left[\begin{array}{c} \tau_{h}\\ f_{h} \end{array}\right]=\left[\begin{array}{cc} \eta & 0\\ \hat{H} & -D \end{array}\right]\left[\begin{array}{c} \frac{\partial {\dot{w}}_{h}}{\partial x}\\ \dot{w_{h}} \end{array}\right]$$

where $\hat{H}=-2HB^{2}l^{2}/3$ in the linear regime with *H* being the linear transfer function of the circuit, and *w*_*h*_, τ_*h*_, and *f*_*h*_ represents the homogenized displacement, stress, and body force fields. Note that this constitutive relationship includes only the viscous part, and the elastic part is not accounted for. Clearly, the parameter $\hat{H}$ correlating the strain rate and the body force appears in the off‐diagonal component of the matrix in Equation (3). We call $\hat{H}$ the Willis‐type viscosity. Note that the other off‐diagonal component (Willis‐type viscosity) is zero because the velocity cannot generate the shear stress. Thus, the coupling of the Willis‐type viscosity displayed by the active wire is asymmetric. By calculating the average power dissipated from or injected into the homogenized active wire due to the asymmetric Willis‐type viscosity under harmonic settings, we find that this average power depends on wave propagation directions (see Supporting Information for details). Thus, if the energy is lost for waves traveling from one direction, the energy is gained for waves traveling from the other direction, indicating that the oscillation amplitude is biased in space.

### 2.4 Continuum Modeling

To quantitatively study the collective self‐oscillation of the active wire, we develop a continuum model. Linear instability is studied first to examine the conditions in which self‐oscillation occurs. We assume *w*_*h*_(*x*, *t*) = *w*_0_ *e* ^*i*(ω*t* − *kx*)^ and derive a linearized characteristic equation as (see Supporting Information)

(4) $$-\rho \omega^{2}+\mathrm{iD}\omega +Tk^{2}+i\eta \omega k^{2}-\hat{H}\omega k=0$$

where the homogenized asymmetric Willis‐type viscosity $\hat{H}=\frac{-2RB^{2}l^{2}}{3\left(-\omega^{2}/\omega_{c}^{2}+i\mu \omega /\omega_{c}+1\right)}$ is defined according to its correlated body force ${\hat{f}}_{h}=\hat{H}\frac{\partial {\dot{w}}_{h}}{\partial x}$. It can be found that the presence of the asymmetric Willis‐type viscosity $\hat{H}$ violates the reciprocity of the active wire. As shown in the last term in Equation (4), when the wavenumber *k* changes the sign, the body force and its work done also change the sign. Thus, the body force behaves differently for waves traveling along different directions. For an under‐damped active wire, the wavenumber *k* can be approximated as

(5) $$k\approx \frac{\hat{H}\omega \pm \sqrt{{\hat{H}}^{2}\omega^{2}+4T\rho \omega^{2}}}{2T}$$

Clearly, when $\hat{H}$ has a nonzero imaginary part, wave amplitudes can no longer be uniform in space. Instead, they are biased along one direction regardless of the wave propagation direction.

Note that when the active wire contains a single unit cell, the active wire is always stable and cannot self‐oscillate no matter how large the ratio is because the control loop is feedforward where body force produces zero strain rate. However, when the active wire contains multiple unit cells, feedback loops are formed as a result of the complex interaction between unit cells. Consequently, the active wire could lose its linear stability and start to self‐oscillate once the amplification ratio is greater than the critical value. Next, by assuming amplification and decaying wave solutions and applying fixed boundary conditions at the two ends of the active wire, we calculate complex eigenfrequencies of the wire (see Supporting Information for details). Figure 3b shows the real and imaginary parts of the eigenfrequencies of the active wire with different electrical amplification ratios *R*. As shown in Figure, when *R* is less than the critical value *R*_*c*_ ≈ 440 A/V, the imaginary part of the eigenfrequency is positive, indicating that the oscillation amplitudes decay with time and the active wire is stable. However, when *R* is greater than the critical value (shaded area), the imaginary part of the eigenfrequency becomes negative so that the oscillation amplitudes amplify exponentially with time and the active wire is unstable. By continuing to increase *R* beyond 1200 A/V, the active wire will enter chaotic oscillations (see Supporting Information for details). The green triangle in the shaded unstable region in Figure 3b indicates the electrical amplification ratio *R* implemented in the active wire demonstrated in Figure 2. We also calculate the mode shapes of displacement and electrical current of the self‐oscillation using the linearized model (see Figure 3c). It can be seen that both the displacement and the actuating current are amplified exponentially in space. The displacement and actuating current have a π/2 phase difference such that energy is constantly pumped into the active wire. Clearly, this model cannot describe the self‐oscillation behavior demonstrated in Figure 2.

Next, we consider saturation nonlinearity to interpret the linear amplification in space. We assume *w*_*h*_(*x*, *t*) = *W*(*x*)sin (*k*_*n*_ *x*)sin (ω*t*), where $k_{n}=\frac{n\pi }{l_{w}}$ and *W*(*x*) is the envelope function. Substituting this equation into two homogenized equations, we derive two leading‐order equations as (see Supporting Information for details)

(6) $$\left(-\omega^{2}\rho W+Tk_{n}^{2}W-T\frac{\partial^{2}W}{\partial x^{2}}\right)\sin\left(k_{n}x\right)=0$$

(7) $$2Tk_{n}\frac{\partial W}{\partial x}\cos\left(k_{n}x\right)=\frac{2}{\mu \pi }BV_{th}\cos\left(k_{n}x\right)$$

As shown in Equation (6), the primary drive of the active wire vanishes, as the current‐related terms are not in sin (*k*_*n*_ *x*) and are not spatially in phase with displacement. On the other hand, the current‐related term *BV*_*th*_cos (*k*_*n*_ *x*) appears in Equation (7) to satisfy the force equilibrium, providing a secondary drive to govern self‐oscillation of the active wire. Solving Equation (7), we find that the envelope *W* is a linear function of *x*, and the self‐oscillation of the active wire is linearly amplified in space. Considering the fact that the self‐oscillation frequency is close to the resonance frequency of a passive wire such that $\omega^{2}\rho \approx Tk_{n}^{2}$, and $\frac{\partial^{2}W}{\partial x^{2}}=0$ due to linear amplification, Equation (6) is satisfied automatically. Note also that since the primary drive is missed, the actuating current is unable to drive oscillations of which the envelope functions are constants. Thus, it is safe to write the envelope function as (see Supporting Information)

(8) $$W=\frac{BV_{th}}{\pi \mu Tk_{n}}x$$

It can be seen that the oscillation amplitude is proportional to the product of the saturation voltage *V*_*th*_ and the strength of the magnetic field *B*, and inversely proportional to the tension force *T* and the wavenumber *k*_*n*_. Figure 3d shows the displacement and actuating current profiles in space, which agrees well with the simulation results in Figure 2c. It is worth mentioning that Figure 2e already demonstrates the linear relationship between the saturation voltage and the oscillation amplitude. In Figure 3e, we examine the relationships between the wavenumber and the amplification slope using the data from Figure 2f (solid dots in the figure). It can be clearly seen that the analytical solutions (dashed curves) agree reasonably well with the simulation results, especially for higher‐order modes at frequencies close to ω_*c*_. This is because, during the derivation of the analytical solution, we assume that the self‐oscillation frequency is equal to the cutoff frequency of the filter (i.e., ω = ω_*c*_).

### 2.5 Standing and Propagating Wave Mode Transition

**Figure 4:**
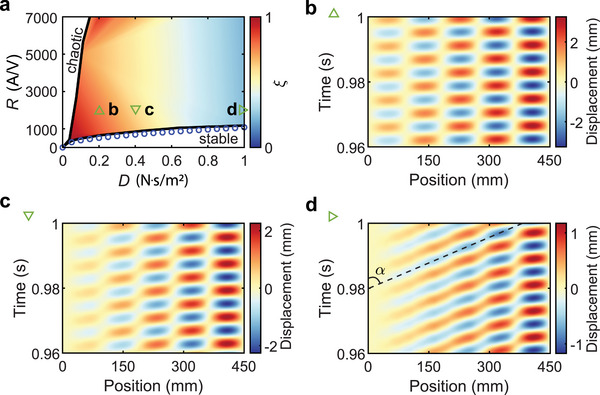
Observation of standing and propagating phase transition when the passive damping of the active wire varies. a) Parameter ξ calculated using different *R* and *D*. The circles are calculated using the linear continuum model, which agree with the stability boundary obtained from the nonlinear time‐dependent simulations. b–d) Displacement profiles on the active wire at different time steps: b) *D* = 0.2 N · s/m^2^ (Standing wave); c) *D* = 0.4 N · s/m^2^ (Standing‐propagating‐mixed wave); d) *D* = 1 N · s/m^2^ (propagating wave with phase velocity *c* = tan α = 21 m/s).

Finally, passive damping also plays an important role in the self‐oscillation of active wires. To see this, we define a parameter $\xi =\frac{w_{max}-w_{min}}{w_{max}+w_{min}}$, where *w*_*max*_ and *w*_*min*_ are the local maximum wave amplitude and its adjacent local minimum wave amplitude, respectively. Consequently, ξ ≈ 1 (*w*_*min*_ ≈ 0) indicates the standing wave mode, and ξ ≈ 0 (*w*_*max*_ ≈ *w*_*min*_) implies the propagating wave mode. We then perform a range of time‐dependent simulations with different damping coefficients and amplification ratios and calculate ξ in **Figure** **4**a. We can see that when *R* is small, the active wire cannot self‐oscillate (the white region labeled “stable”). On the other hand, when *D* is small, the active wire quickly enters chaotic states (another white region labeled “chaotic”). The large colored region in the figure denotes the self‐oscillation with a single frequency. Interestingly, in this region, we find that the standing wave mode gradually transforms to a propagating wave mode as the damping coefficient *D* increases. Figure 4b–d illustrates the displacement profiles for standing, standing‐propagating‐mixed, and propagating waves, in time and spacial domains, respectively. This unusual behavior can be interpreted by considering two propagating modes traveling in opposite directions: One is amplified and the other is decayed. When *D* is small, both the decaying and amplification modes exist in the entire active wire, although the amplitude of the decaying mode is small at the end of the wire. This co‐existence gives rise to the standing wave mode. However, when *D* becomes large, the decaying mode damps out quickly, and only the amplification mode remains, leading to the propagating mode.

## 3 Conclusion

In this work, we suggest a self‐oscillation strategy enabled by asymmetric Willis viscosity. We realize the asymmetric Willis viscosity using active wires and reveal the physical principles underpinning the biased self‐oscillation with linear envelope functions. We show the capability of independent tailoring of self‐oscillation frequencies and mode shapes as well as standing propagating mode transitions. The studies presented in this work focus on the design, numerical simulations, and theoretical understanding of the active wire. It is still worth mentioning that the active wire can be realized and tested in experiments, which will involve a range of well‐planned procedures, from wire and circuit fabrication and setup to signal and vibration tests and measurements (see our detailed discussion in Supporting Information). Overall, this work can benefit the understanding of nonstandard self‐oscillation systems and prompt the design of autonomous materials, e.g., motors, agitators, pumps, and shakers.

## Conflict of Interest

All authors declare no competing interests.

## Author Contributions

X.P. and X.H. conducted numerical experiments and analytical studies; Y.C. conceived the concept, initiated the studies, and supervised the research; All the authors discussed the results; All authors wrote the manuscript and interpreted the results.

## Supporting information

Supporting Information

## Data Availability

The data that support the findings of this study are available from the corresponding author upon reasonable request.
