# Supplementary material for: Self‐Oscillation in Active Wires with Asymmetric Willis‐Type Viscosity
Source: Adv Sci (Weinh). 2025 Apr 17;12(23):2500737. doi: 10.1002/advs.202500737 (PMC12199388; doi:10.1002/advs.202500737)
Supplement: Supplementary file 1 — Supporting Information [file ADVS-12-2500737-s001.pdf]

ADVANCED SCIENCE
Open Access

Supporting Information

for *Adv. Sci.*, DOI 10.1002/advs.202500737

Self-Oscillation in Active Wires with Asymmetric Willis-Type Viscosity

*Xingbo Pu, Xiaoyu Hou, Antonio Palermo and Yangyang Chen**

Supporting Information for Self-oscillation in Active Wires with Asymmetric Willis-type Viscosity

Xingbo Pu,[1] Xiaoyu Hou,[1] Antonio Palermo,[2] and Yangyang Chen[1,*]

[1] *Department of Mechanical and Aerospace Engineering, The Hong Kong University of Science and Technology, Clear Water Bay, Kowloon, Hong Kong*

[2] *Department of Civil, Chemical, Environmental and Materials Engineering, University of Bologna, 40136 Bologna, Italy*

Contents

S1. Numerical experiment details 2

S2. Self-oscillations in 3D space 2

S3. Effects of initial velocity 2

S4. Chaotic self-oscillation of the active wire 3

S5. Energy distribution of the active wire 4

S6. Asymmetric Willis-type viscosity 4

S7. Continuum model 5

S8. Detailed description of the unidirectional amplification 6

S9. Detailed derivations of the linear amplification in space 7
A. Undamped active wire 7
B. Damped active wire 8

S10. Discrete model 9

S11. Linearization of the discrete model 11

S12. Effects of the wire diameter on self-oscillations 12

S13. Potential Experimental Realization 12

References 13

---

*Electronic address: maeychen@ust.hk

S1. NUMERICAL EXPERIMENT DETAILS

Since the length-to-radius ratio of the wire is extremely large in the design, the equation of motion for the active wire can be well described by a simple string model. Compared to a full three-dimensional (3D) model, the one-dimensional string model achieves an optimal balance between computational efficiency and accuracy [1]. In this work, we assume that the wire moves in the vertical plane (a condition that has also been validated in 3D simulations), which allows us to model the vibration of the active wire using the one-dimensional classical wave equation. To perform numerical simulations, we combine the modulus of the partial differential equation (PDE) interface with Ordinary Differential Equations in a one-dimensional setting in COMSOL Multiphysics to solve Eqs. 1 and 2 in the main text. For simulations, fixed boundary conditions (clamped ends, $w = 0$) are chosen to replicate real-world applications where wires are anchored at both ends (e.g., cables in bridges, tensegrity systems, or robotic tendons). The wire is discretized using a convergent mesh of Lagrange quadratic elements and time-dependent studies are performed using the generalized alpha method.

S2. SELF-OSCILLATIONS IN 3D SPACE

To see if the wire exhibits rotational effects in addition to its movement in the z-direction, we model the wire's self-oscillation with displacement fields in a three-dimensional (3D) space using COMSOL Multiphysics. In this model, the wire is permitted to have displacements in the x-direction (u), the y-direction (v) and the z-direction (w). We assume that the magnetic field B is perpendicular to the x-axis and forms an angle θ with the y-axis. As illustrated in Fig. S1a, when current flows through the wire, the resulting magnetic force $\hat{f}_h$ is perpendicular to the wire and forms an angle θ with the z-axis. Consequently, the wire should maintain self-oscillation within a plane inclined at an angle θ to the z-axis without any rotational effects. To verify this, we first consider the case where $\theta = 0$. In this configuration, we expect results identical to those shown in Figure 2c of the main text, as the body force component in the y-direction is zero. Using parameters from Figure 2 of the main text, Fig. S1b displays the displacement fields of the entire wire at two time steps when they reach their maximum and/or minimum values, which indeed match those shown in Figure 2c of the main text. Additionally, we consider $\theta = -\pi/6$. In this scenario, we anticipate that the wire's self-oscillation occurs in a plane inclined at an angle θ to the z-axis. Fig. S1c confirms this expectation, showing that the wire oscillates within the anticipated plane (shaded area).

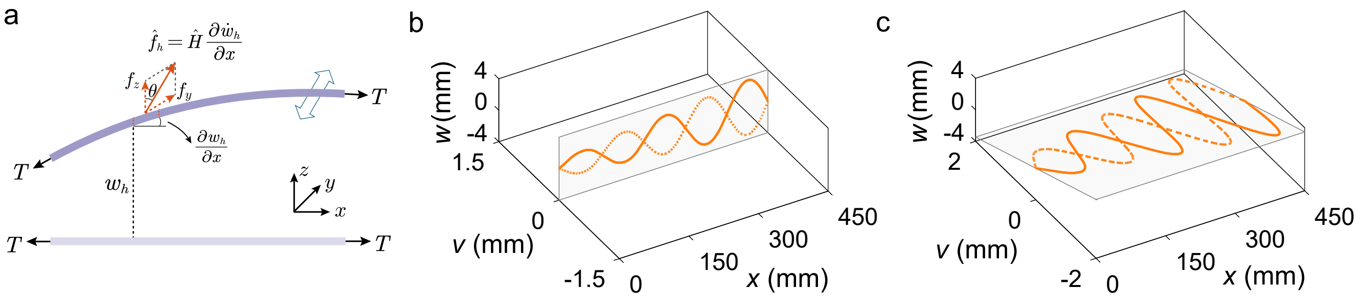

Fig. S1: Self-oscillation in 3D space. (a) Homogenized model. The magnetic field B is oriented perpendicular to the x-axis and forms an angle of θ with respect to the y-axis. Displacement fields on the entire wire at two-time steps when they reach maximum and/or minimum values for (b) $\theta = 0$ and (c) $\theta = -\pi/6$.

S3. EFFECTS OF INITIAL VELOCITY

Using parameters $\rho = 4.5$ g/m (wire diameter: 0.8 mm), $T = 2$ N, $D = 0.15$ N $\cdot$ s/m^2, $\eta = 0$, $B = 1$ T, $\omega_c = 700$ rad/s, $\mu = 0.5$, $l = 10$ mm, $R = 1000$ A/V, and the total length of the active wire $l_w = 0.45$ m, we show in Fig. S2 the time histories of displacement and actuating current at the midpoint of the rightmost unit cell for two distinct initial velocities: 0.1 m/s (Fig. S2a,b) and 0.001 m/s (Fig. S2c,d). It can be observed that for the smaller initial velocity, the active wire takes a longer time to reach stable self-oscillation, and the evolution during this process becomes smoother. However, the limit-cycle oscillation remains invariant when the initial conditions change.

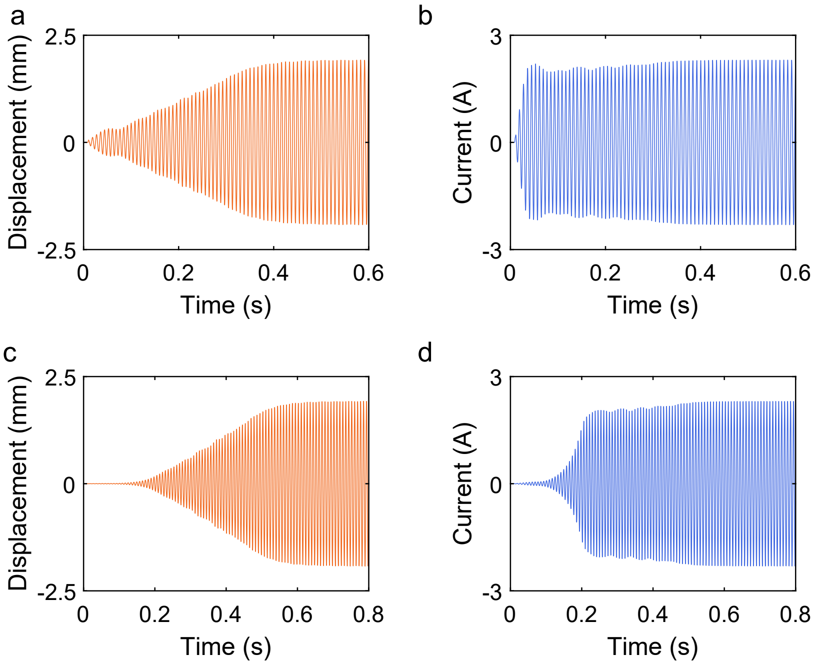

Fig. S2: Effects of initial velocity on the self-oscillation of the active wire. Displacement and actuating current evolved in time at the midpoint of the rightmost unit cell for two distinct initial velocities: 0.1 m/s (a,b) and 0.001 m/s (c,d).

S4. CHAOTIC SELF-OSCILLATION OF THE ACTIVE WIRE

By increasing the amplification ratio R above 1.2×10^3 A/V, the biased single-frequency self-oscillation no longer exists (see Fig. S3a). We find that when 1.2×10^3 A/V$\leq R \leq 7.0 \times 10^3$ A/V three oscillation frequencies appear, which remain nearly unchanged in this region. However, when $R > 7.0 \times 10^3$ A/V, even more numbers of oscillation frequencies emerge, which are largely unpredictable and distributed irregularly, indicating that the active wire enters to chaotic states (see the gray area in Fig. S3b). Consequently, the phase portrait and the displacement profile also become unpredictable as shown in Figs. S3c,d.

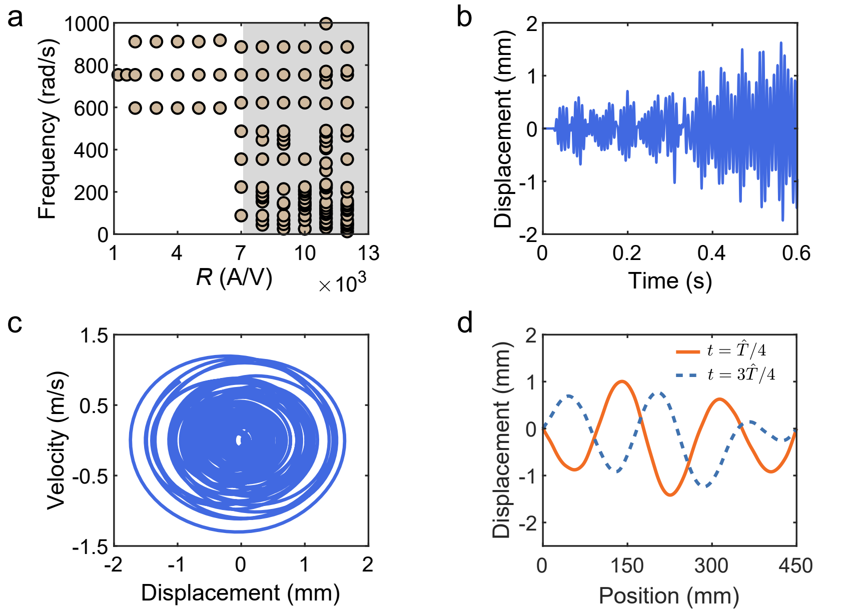

Fig. S3: Chaotic self-oscillation of the active wire. (a) Self-oscillation frequencies with different R. (b) Displacement in function of time at the center of the wire. (c) Phase portrait between displacement and velocity at the center of the wire. (d) Displacement profiles on the entire wire. (b-d) $R = 10.0 \times 10^3$ A/V.

S5. ENERGY DISTRIBUTION OF THE ACTIVE WIRE

In the absence of nonlinearity, energy continues to pump into the active wire as time progresses (see the solid curve in Fig. S4a), giving rise to the exponential amplifications of both displacement and current in Figure 3c of the main text. By contrast, when V_{th} becomes a finite positive number, the nonlinearity of the saturation circuit emerges, leading to the limit-cycle self-oscillation of the active wire shown in Figure 2 of the main text, where the average power gained externally by the active wire is equal to the average power dissipated internally due to damping (see Fig. S4b). By continuing to increase R beyond 1200 A/V, the active wire will enter chaotic oscillations, where the energy evolution becomes unpredictable as well (see Fig. S4c).

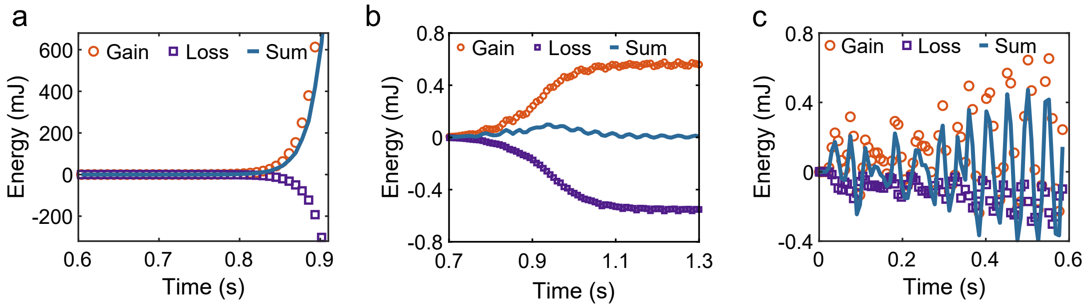

Fig. S4: Average energy injected and lost during self-oscillation. (a) $R = 0.45 \times 10^3$ A/V (linear model); (b) $R = 1.0 \times 10^3$ A/V (limit-cycle oscillation); (c) $R = 10.0 \times 10^3$ A/V (chaotic).

S6. ASYMMETRIC WILLIS-TYPE VISCOSITY

We separate the Willis-type viscosity appearing in Equation (3) of the main text into a symmetric tensor and an antisymmetric tensor, and calculate the average power dissipated from or injected into the active wire. We found that the average powers are equal to $\frac{1}{2}|\hat{H}|W_t\Gamma_t \cos\phi \cos\varphi$ and $\frac{1}{2}|\hat{H}|W_t\Gamma_t \sin\phi \sin\varphi$ for the symmetric and antisymmetric Willis-type viscosity, respectively. In the equation, W_t, Γ_t, ϕ, and φ represent the amplitude of the velocity, the

amplitude of the strain rate, the phase difference between the velocity and the strain rate, and the phase delay caused by $\hat{H}$.

As shown in Figure 2f of the main text, the oscillation frequency is close to the cutoff frequency of the circuit such that the phase delay φ is around $-\frac{\pi}{2}$ and the power induced by the symmetric Willis-type viscosity $\frac{1}{2}|\hat{H}|W_t\Gamma_t\cos\phi\cos\varphi$ can be ignored. Examining the power generated by the antisymmetric Willis-type viscosity $\frac{1}{2}|\hat{H}|W_t\Gamma_t\sin\phi\sin\varphi$, we make two fundamental observations relevant to self-oscillation: (i) The power is proportional to $\sin\phi$, which indicates that if the energy is lost for waves traveling from one direction ($\phi\approx\pm\frac{\pi}{2}$), the energy is gained for waves traveling from the other direction ($\phi\approx\mp\frac{\pi}{2}$). The standing wave shown in Figure 2c can be decomposed into two propagating waves with the same amplitudes but traveling in opposite directions. As a result, the net power is zero, but the flow of energy remains, which biases the oscillation mode shapes to allow them to amplify along one direction. The spatial amplification ratio is then controlled by the magnitude of the antisymmetric Willis-type viscosity. (ii) The power is also proportional to $\sin\varphi$. As a matter of fact, self-oscillation occurs at a frequency where energy injection reaches its maximum, that is $\varphi\approx\pm\frac{\pi}{2}$, which coincides with the finding in Figure 2f. Thus, we can leverage the phase angle of $\hat{H}$ at different frequencies to control the self-oscillation frequency. Together, it implies that self-oscillation frequencies and mode shapes can be independently tailored by the antisymmetric Willis-type viscosity.

S7. CONTINUUM MODEL

To calculate the eigenfrequencies of a finite active wire, we derive an analytical model at the continuum limit. We first calculate the sensing voltage

$$\bar{V}_s=\left(\int_{\ell_1}\frac{\partial w}{\partial t}\mathrm{d}x-\int_{\ell_2}\frac{\partial w}{\partial t}\mathrm{d}x\right)B\approx-2Bl^2\frac{\partial^2 w_h}{\partial x\partial t},\tag{S1}$$

where w_h represents the homogenized displacement, ℓ_1 and ℓ_2 denote the lengths of the left- and right-hand sensors, respectively. We assume that the control circuit is distributed locally at the continuum limit so that the homogenized actuating current is governed by

$$\frac{1}{\omega_c^2}\ddot{I}+\frac{\mu}{\omega_c}\dot{I}+I=\begin{cases}\hat{R}B\frac{\partial^2 w_h}{\partial x\partial t}, & |\hat{R}B\frac{\partial^2 w_h}{\partial x\partial t}|<V_{th}\\ \mathrm{sgn}(\hat{R}B\frac{\partial^2 w_h}{\partial x\partial t})V_{th}, & |\hat{R}B\frac{\partial^2 w_h}{\partial x\partial t}|\geq V_{th}\end{cases}\tag{S2}$$

where $\hat{R}=-2Rl^2/3$. Implementing the classical string model, the motion of the active wire can be described by

$$\rho\frac{\partial^2 w_h}{\partial t^2}+D\frac{\partial w_h}{\partial t}-T\frac{\partial^2 w_h}{\partial x^2}-BI=0.\tag{S3}$$

Note that we ignore the viscosity coefficient η and the nonlinear axial deformations of the active wire in Eq. (S3). To find eigenfrequencies of the finite active wire, we first linearize Eq. (S2) as

$$\frac{1}{\omega_c^2}\ddot{I}+\frac{\mu}{\omega_c}\dot{I}+I=\hat{R}B\frac{\partial^2 w_h}{\partial x\partial t}.\tag{S4}$$

We then assume wave solutions for the displacement w_h and the current I as

$$w_h(x,t)=w_0e^{i(\omega t-kx)},\tag{S5a}$$

$$I(x,t)=I_0e^{i(\omega t-kx)},\tag{S5b}$$

where k, w_0, and I_0 represent the wavenumber, amplitudes of the displacement and the current, respectively. Substituting Eqs. (S5a) and (S5b) into Eqs. (S3) and (S4), we obtain a characteristic equation which reads as

$$-\rho\omega^2+iD\omega+Tk^2-\hat{H}\omega k=0,\tag{S6}$$

where $\hat{H}=\frac{\hat{R}B^2}{-\omega^2/\omega_c^2+i\mu\omega/\omega_c+1}$ is the homogenized asymmetric Willis-type viscosity. Clearly, given the frequency ω in Eq. (S6), two wavenumbers can be attained from the polynomial equation of the second degree as

$$k_{1,2}=\frac{1}{2T}\left(\frac{\hat{R}B^2\omega}{-\omega^2/\omega_c^2+i\mu\omega/\omega_c+1}\pm\sqrt{\left(\frac{\hat{R}B^2\omega}{-\omega^2/\omega_c^2+i\mu\omega/\omega_c+1}\right)^2-4T(-\rho\omega^2+iD\omega)}\right).\tag{S7}$$

As indicated in Eq. (S7), the two wavenumbers can be different in both real and imaginary parts depending on the second term of the equation. Consequently, the left- and right-propagating waves can have different wavelengths and/or decaying depths. In particular, waves traveling from one direction are decayed; waves traveling from the other direction can be amplified (see detailed discussion in the next section).

The solution of the displacement is the combination of the two wave equations given by

$$w_h(x,t) = w_1 e^{i(\omega t - k_1 x)} + w_2 e^{i(\omega t - k_2 x)}, \tag{S8}$$

where w_1 and w_2 are the amplitudes of the two waves. Considering fixed boundary conditions at the ends of the active wire $w_h(x=0) = w_h(x=l_w) = 0$, we can obtain

$$w_1 + w_2 = 0, \tag{S9a}$$

$$w_1 e^{-ik_1 l_w} + w_2 e^{-ik_2 l_w} = 0. \tag{S9b}$$

Combining Eqs. (S9a) and (S9b) leads to

$$k_1 - k_2 = \frac{2n\pi}{l_w} = \frac{1}{T}\sqrt{\left(\frac{\hat{R}B^2\omega}{-\omega^2/\omega_c^2 + i\mu\omega/\omega_c + 1}\right)^2 - 4T(-\rho\omega^2 + iD\omega)}, \quad n = 0, 1, 2, ... \tag{S10}$$

Eq. (S10) can be rewritten into a polynomial equation as

$$a_6\omega^6 + a_5\omega^5 + \cdots + a_1\omega + a_0 = 0, \tag{S11}$$

where

$$a_6 = 4T\rho/\omega_c^4, \tag{S12a}$$
$$a_5 = -4iDT/\omega_c^4 - 8iT\rho\mu/\omega_c^3, \tag{S12b}$$
$$a_4 = -8DT\mu/\omega_c^3 - (2n\pi T/l_w)^2/\omega_c^4 - 4T\rho\mu^2/\omega_c^2 - 8T\rho/\omega_c^2, \tag{S12c}$$
$$a_3 = 4iDT\mu^2/\omega_c^2 + 8iDT/\omega_c^2 + 8i(n\pi T/l_w)^2\mu/\omega_c^3 + 8iT\rho\mu/\omega_c, \tag{S12d}$$
$$a_2 = 8DT\mu/\omega_c + (2n\pi T/l_w)^2\mu^2/\omega_c^2 + 8(n\pi T/l_w)^2/\omega_c^2 + \hat{R}^2B^4 + 4T\rho, \tag{S12e}$$
$$a_1 = -4iDT - 8i(n\pi T/l_w)^2\mu/\omega_c, \tag{S12f}$$
$$a_0 = -(2n\pi T/l_w)^2. \tag{S12g}$$

Eigenfrequencies of the finite active wire are the roots of the polynomial equation in Eq. (S11). If there exists a pair of eigenfrequencies whose imaginary parts are negative, both the displacement and the current are amplified exponentially in time and self-oscillation occurs. Further, it is of interest to identify the conditions of the stability boundary where $\mathrm{Im}(\omega) = 0$. Enforcing this equation in Eq. (S10) gives rise to

$$(\hat{R}B^2\omega)^2(\omega^2/\omega_c^2 - 1)\mu/\omega_c = 2TD[(\mu\omega/\omega_c)^2 + (\omega^2/\omega_c^2 - 1)^2]^2. \tag{S13}$$

Clearly, at the stability boundary, the following relationships should be satisfied

$$|\hat{R}| \propto \sqrt{D}, \quad |\hat{R}| \propto \sqrt{T}, \quad \omega > \omega_c. \tag{S14}$$

S8. DETAILED DESCRIPTION OF THE UNIDIRECTIONAL AMPLIFICATION

To show the unidirectional amplification of self-oscillation in the active wire using wavenumbers, we simplify Eq. (S7) at the self-oscillation frequency. In particular, we consider that the self-oscillation frequency is approximately equal to the cut-off frequency of the electrical filter $\omega \approx \omega_c$, and ignore the passive damping D of the wire. Then, Eq. (S7) can be reduced to

$$k_{1,2} = \frac{\omega_c}{2T}\left(-\frac{i\hat{R}B^2}{\mu} \pm \sqrt{4T\rho - \frac{\hat{R}^2B^4}{\mu^2}}\right). \tag{S15}$$

To ensure wave solutions, we focus on the cases where $\hat{R}^2B^4/\mu^2 < 4T\rho$. Thus, when $\hat{R} < 0$, the imaginary parts of the two wavenumbers are positive, and the displacement amplitude is amplified along the positive x direction (see the left panel in Fig. S5). On the contrary, when $\hat{R} > 0$, the imaginary parts of the two wavenumbers become negative, and the displacement amplitude is amplified along the negative x direction (see the right panel in Fig. S5).

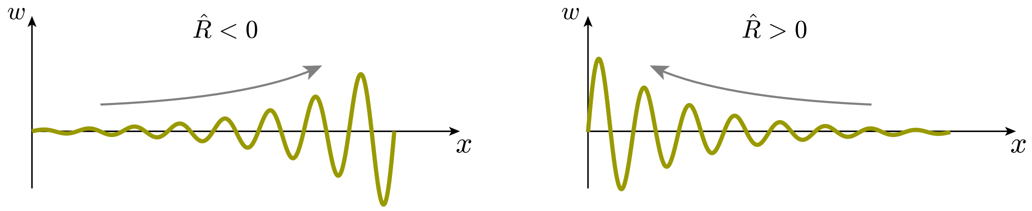

Fig. S5: Displacement profiles of unidirectional amplification of the active wire.

S9. DETAILED DERIVATIONS OF THE LINEAR AMPLIFICATION IN SPACE

In this section, we list detailed analytical derivations to explain linear amplification in space. We first propose a simplified model for the active wire without passive damping and then extend this simplified model to the active wire in the presence of passive damping.

A. Undamped active wire

In the absence of passive damping ($D = 0$), we substitute the ansatz $w_h(x,t) = W(x)\sin(k_nx)\sin(\omega t)$ with $k_n = \frac{n\pi}{l_w}$ into Eqs. (S2) and (S3) to obtain two equations of motion for $W(x)$ and $I(x)$ as

$$(-\omega^2\rho W + Tk_n^2W - T\frac{\partial^2 W}{\partial x^2})\sin(k_nx)\sin(\omega t) - 2Tk_n\frac{\partial W}{\partial x}\cos(k_nx)\sin(\omega t) = BI, \tag{S16}$$

$$\frac{1}{\omega_c^2}\ddot{I} + \frac{\mu}{\omega_c}\dot{I} + I = \begin{cases} \hat{R}B\omega\left(\frac{\partial W}{\partial x}\sin(k_nx) + Wk_n\cos(k_nx)\right)\cos(\omega t), \\ \qquad \text{if } |\hat{R}B\omega\left(\frac{\partial W}{\partial x}\sin(k_nx) + Wk_n\cos(k_nx)\right)\cos(\omega t)| < V_{th} \\ \operatorname{sgn}\left[\hat{R}B\omega\left(\frac{\partial W}{\partial x}\sin(k_nx) + Wk_n\cos(k_nx)\right)\cos(\omega t)\right]V_{th}, \\ \qquad \text{if } |\hat{R}B\omega\left(\frac{\partial W}{\partial x}\sin(k_nx) + Wk_n\cos(k_nx)\right)\cos(\omega t)| \geq V_{th} \end{cases} \tag{S17}$$

Note that it is extremely difficult to obtain exact analytical solutions for Eqs. (S16) and (S17). To seek approximate solutions, we first neglect the term $\frac{\partial W}{\partial x}\sin(k_nx)$ in Eq. (S17), since the gradient of the envelope function $\frac{\partial W}{\partial x}$ is much less dominant than Wk_n. Next, we calculate the Fourier series of the right-hand side function in Eq. (S17), and keep only the terms of the first order. Eq. (S17) becomes

$$\frac{1}{\omega_c^2}\ddot{I} + \frac{\mu}{\omega_c}\dot{I} + I \approx -\frac{2V_{th}}{\pi}\cos(k_nx)\cos(\omega t). \tag{S18}$$

Finally, we assume that the self-oscillation frequency is close to the cutoff frequency of the electrical filter $\omega \approx \omega_c$ such that

$$I = -\frac{2}{\mu\pi}V_{th}\cos(k_nx)\sin(\omega t). \tag{S19}$$

Combining Eqs. (S16) and (S19) leads to

$$(-\omega^2\rho W + Tk_n^2W - T\frac{\partial^2 W}{\partial x^2})\sin(k_nx)\sin(\omega t) - (2Tk_n\frac{\partial W}{\partial x} - \frac{2}{\mu\pi}BV_{th})\cos(k_nx)\sin(\omega t) = 0. \tag{S20}$$

Eq. (S20) can be written into two separate equations as

$$\left(-\omega^2\rho W + Tk_n^2 W - T\frac{\partial^2 W}{\partial x^2}\right)\sin\left(k_n x\right) = 0, \tag{S21}$$

$$2Tk_n\frac{\partial W}{\partial x}\cos\left(k_n x\right) = \frac{2}{\mu\pi}BV_{th}\cos\left(k_n x\right). \tag{S22}$$

Considering the fact that the self-oscillation frequency is also close to the resonant frequency of the wire $\omega^2\rho \approx Tk_n^2$, Eqs. (S21) and (S22) can be further reduced to

$$\frac{\partial^2 W}{\partial x^2} = 0, \tag{S23}$$

$$\frac{\partial W}{\partial x} = \frac{BV_{th}}{\pi\mu Tk_n}. \tag{S24}$$

According to the discussion made in the main text, the mode shape function can be expressed as

$$w_h(x) = \frac{BV_{th}}{\pi\mu Tk_n}\cdot x\sin(k_n x). \tag{S25}$$

To validate the analytical model, Fig. S6 shows the mode shape functions obtained from numerical simulations (blue circles) and Eq. (S25) (brown curve). Good agreement is clearly seen in the figure. However, note also that discrepancies exist for $0 < x < 200$ mm where the term $\frac{\partial W}{\partial x}\sin(k_n x)$ shows non-negligible effects.

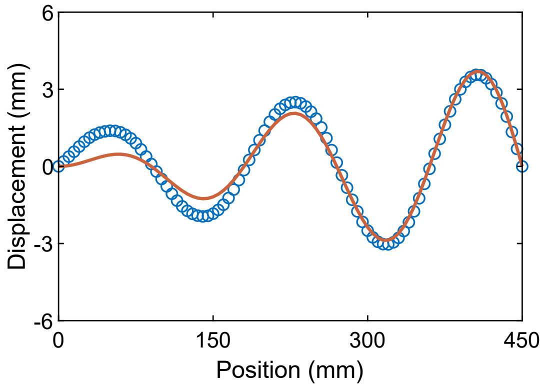

Fig. S6: Mode shape functions obtained from numerical simulations (blue circles) and Eq. (S25) (brown curve).

B. Damped active wire

In the presence of passive damping D, the assumption $w_h(x,t) = W(x)\sin(k_n x)\sin(\omega t)$ made in the previous section will no longer satisfy the two equations of motion. To still solve the two equations analytically, we generalize the assumption by enforcing $w_h(x,t) = W_1(x)\sin(k_n x)\sin(\omega t) + W_2(x)\sin(k_n x)\cos(\omega t)$ and substitute it into Eqs. (S2) and (S3), which leads to

$$\begin{aligned}(-\omega^2\rho W_1 + Tk_n^2 W_1 - D\omega W_2 - T\frac{\partial^2 W_1}{\partial x^2})\sin(k_n x)\sin(\omega t) - 2Tk_n\frac{\partial W_1}{\partial x}\cos(k_n x)\sin(\omega t)\\ -(\omega^2\rho W_2 - Tk_n^2 W_2 - D\omega W_1 + T\frac{\partial^2 W_2}{\partial x^2})\sin(k_n x)\cos(\omega t) - 2Tk_n\frac{\partial W_2}{\partial x}\cos(k_n x)\cos(\omega t) = BI,\end{aligned} \tag{S26}$$

$$\frac{1}{\omega_c^2}\ddot{I} + \frac{\mu}{\omega_c}\dot{I} + I = \begin{cases}\Xi, & |\hat{R}B\frac{\partial^2 w_h}{\partial x\partial t}| < V_{th}\\ \mathrm{sgn}(\Xi)V_{th}, & |\hat{R}B\frac{\partial^2 w_h}{\partial x\partial t}| \geq V_{th}\end{cases} \tag{S27}$$

where

$$\Xi = \hat{R}B\omega[(\frac{\partial W_1}{\partial x}\sin(k_n x) + W_1 k_n \cos(k_n x))\cos(\omega t) - (\frac{\partial W_2}{\partial x}\sin(k_n x) + W_2 k_n \cos(k_n x))\sin(\omega t)]. \tag{S28}$$

Similarly, to seek approximate solutions, we neglect $\frac{\partial W_1}{\partial x}\sin(k_n x)$ and $\frac{\partial W_2}{\partial x}\sin(k_n x)$ in the first and second terms of Eq. (S27). We also assume that the self-oscillation frequency is close to the cutoff frequency of the electrical filter $\omega \approx \omega_c$. Performing the same procedures as those done in the previous section, the solution of Eq. (S27) can be approximated by

$$I \approx -\frac{2V_{th}}{\pi\mu}\cos(k_n x)\cos(\omega t) - \frac{2V_{th}}{\pi\mu}\cos(k_n x)\sin(\omega t). \tag{S29}$$

Combining Eqs. (S26) and (S29) leads to

$$(-\omega^2 \rho W_1 + Tk_n^2 W_1 - D\omega W_2 - T\frac{\partial^2 W_1}{\partial x^2})\sin(k_n x)\sin(\omega t) = 0, \tag{S30a}$$

$$(-\omega^2 \rho W_2 + Tk_n^2 W_2 + D\omega W_1 - T\frac{\partial^2 W_2}{\partial x^2})\sin(k_n x)\cos(\omega t) = 0, \tag{S30b}$$

$$(-2Tk_n\frac{\partial W_1}{\partial x} + \frac{2}{\mu\pi}BV_{th})\cos(k_n x)\sin(\omega t) = 0, \tag{S30c}$$

$$(-2Tk_n\frac{\partial W_2}{\partial x} + \frac{2}{\mu\pi}BV_{th})\cos(k_n x)\cos(\omega t) = 0. \tag{S30d}$$

It can be observed from Eqs. (S30c) and (S30d) that the saturated nonlinear circuit is the source of power that prompts the self-oscillation characterized by a linear envelope function with the slope

$$\frac{\partial W_1}{\partial x} = \frac{\partial W_2}{\partial x} = \frac{BV_{th}}{\pi\mu T k_n}. \tag{S31}$$

The conclusions attained from the analysis of the damped active wire actually coincide with those derived from the analysis of the undamped active wire.

S10. DISCRETE MODEL

In this section, we develop a discrete mass-spring model to validate the time-dependent finite element (FE) simulations performed in the main text. To model the dynamic behaviors of the active wire, we implement a one-dimensional mass-spring chain shown in Fig. S7a. It is worth mentioning that although the discrete masses of the chain move horizontally other than vertically in the active wire, the discrete chain shares the same physical principle as the active wire. Thus, the numerical results attained from the discrete chain apply directly to the continuum active wire. To build the unit cell of the discrete chain, we employ three masses with identical weight $m_0 = \rho l$ connected by three linear springs $k_0 = T/l$. Three dashpots with the coefficient being $c_0 = Dl$ are installed between the masses and the ground. Similar to the active wire, the velocities of the left- and right-hand side masses are measured and input into a controller that generates a body force applied to the middle mass. The control follows the same law as we propose for the active wire. Thus, the equation of motion of the n-th unit cell reads

$$m_0\ddot{u}_n^{(1)} + c_0\dot{u}_n^{(1)} - k_0\left(u_{n-1}^{(3)} + u_n^{(2)} - 2u_n^{(1)}\right) = 0, \tag{S32a}$$

$$m_0\ddot{u}_n^{(2)} + c_0\dot{u}_n^{(2)} - k_0\left(u_n^{(1)} + u_n^{(3)} - 2u_n^{(2)}\right) - q_n = 0, \tag{S32b}$$

$$m_0\ddot{u}_n^{(3)} + c_0\dot{u}_n^{(3)} - k_0\left(u_n^{(2)} + u_{n+1}^{(1)} - 2u_n^{(3)}\right) = 0, \tag{S32c}$$

$$\frac{1}{\omega_c^2}\ddot{q}_n + \frac{\mu}{\omega_c}\dot{q}_n + q_n = \begin{cases} R_q\left(\dot{u}_n^{(1)} - \dot{u}_n^{(3)}\right), & |R_q\left(\dot{u}_n^{(1)} - \dot{u}_n^{(3)}\right)| < Q_{th}, \\ \operatorname{sgn}\left[R_q\left(\dot{u}_n^{(1)} - \dot{u}_n^{(3)}\right)\right] Q_{th}, & |R_q\left(\dot{u}_n^{(1)} - \dot{u}_n^{(3)}\right)| \geq Q_{th}, \end{cases} \tag{S33}$$

where $u_n^{(i)}$, q_n, $R_q = R(Bl)^2$, and $Q_{th} = BlV_{th}$ denote the displacement of the i-th mass in the n-th unit cell, the active body force applied on the middle mass, the amplification ratio between the velocity and the active body force, and the body force threshold, respectively. To study the dynamics of a finite active wire, we write the equations of motion of all the masses into a matrix form as

$$\mathbf{M}_{3N\times3N}\ddot{\mathbf{u}} + \mathbf{C}_{3N\times3N}\dot{\mathbf{u}} + \mathbf{K}_{3N\times3N}\mathbf{u} = \mathbf{Q}, \tag{S34}$$

$$\mathbf{m}_{N\times N}\ddot{\mathbf{q}} + \mathbf{c}_{N\times N}\dot{\mathbf{q}} + \mathbf{k}_{N\times N}\mathbf{q} = \mathbf{f}, \tag{S35}$$

where N is the number of the unit cells of the finite active wire, and other matrices in the equation are written as

$$\mathbf{M} = \rho l \begin{bmatrix} 1 & 0 & 0 & \dots & 0 \\ 0 & 1 & 0 & \dots & 0 \\ \vdots & \vdots & \vdots & \ddots & \vdots \\ 0 & 0 & 0 & \dots & 1 \end{bmatrix}, \mathbf{C} = Dl \begin{bmatrix} 1 & 0 & 0 & \dots & 0 \\ 0 & 1 & 0 & \dots & 0 \\ \vdots & \vdots & \vdots & \ddots & \vdots \\ 0 & 0 & 0 & \dots & 1 \end{bmatrix}, \mathbf{K} = \frac{T}{l} \begin{bmatrix} 3 & -1 & 0 & \dots & 0 \\ -1 & 2 & -1 & \dots & 0 \\ \vdots & \vdots & \vdots & \ddots & \vdots \\ 0 & 0 & 0 & \dots & 3 \end{bmatrix}, \mathbf{u} = \begin{bmatrix} u_1^{(1)} \\ u_1^{(2)} \\ \vdots \\ u_N^{(3)} \end{bmatrix}, \mathbf{Q} = \begin{bmatrix} \mathbf{q}_1 \\ \mathbf{q}_2 \\ \vdots \\ \mathbf{q}_N \end{bmatrix}, \tag{S36}$$

$$\mathbf{m} = \frac{1}{\omega_c^2} \begin{bmatrix} 1 & 0 & 0 & \dots & 0 \\ 0 & 1 & 0 & \dots & 0 \\ \vdots & \vdots & \vdots & \ddots & \vdots \\ 0 & 0 & 0 & \dots & 1 \end{bmatrix}, \quad \mathbf{c} = \frac{\mu}{\omega_c} \begin{bmatrix} 1 & 0 & 0 & \dots & 0 \\ 0 & 1 & 0 & \dots & 0 \\ \vdots & \vdots & \vdots & \ddots & \vdots \\ 0 & 0 & 0 & \dots & 1 \end{bmatrix}, \quad \mathbf{k} = \begin{bmatrix} 1 & 0 & 0 & \dots & 0 \\ 0 & 1 & 0 & \dots & 0 \\ \vdots & \vdots & \vdots & \ddots & \vdots \\ 0 & 0 & 0 & \dots & 1 \end{bmatrix}, \quad \mathbf{q} = \begin{bmatrix} q_1 \\ q_2 \\ \vdots \\ q_n \end{bmatrix}, \quad \mathbf{f} = \begin{bmatrix} f_1 \\ f_2 \\ \vdots \\ f_n \end{bmatrix}, \tag{S37}$$

$$\mathbf{q}_n = (0, q_n, 0)^T, \quad n = 1, 2, \cdots, N. \tag{S38}$$

$$f_n = R_q(\dot{u}_n^{(1)} - \dot{u}_n^{(3)})(|R_q(\dot{u}_n^{(1)} - \dot{u}_n^{(3)})| < Q_{th}) + Q_{th} \cdot \operatorname{sgn}[R_q(\dot{u}_n^{(1)} - \dot{u}_n^{(3)})](|R_q(\dot{u}_n^{(1)} - \dot{u}_n^{(3)})| \geq Q_{th}). \tag{S39}$$

Next, we reorganize the two equations of motion into one as

$$\underbrace{\begin{bmatrix} \mathbf{M}_{3N\times3N} & \mathbf{0}_{3N\times N} \\ \mathbf{0}_{N\times3N} & \mathbf{m}_{N\times N} \end{bmatrix}}_{\bar{\mathbf{M}}} \begin{bmatrix} \ddot{\mathbf{u}} \\ \ddot{\mathbf{q}} \end{bmatrix} + \underbrace{\begin{bmatrix} \mathbf{C}_{3N\times3N} & \mathbf{0}_{3N\times N} \\ \mathbf{0}_{N\times3N} & \mathbf{c}_{N\times N} \end{bmatrix}}_{\bar{\mathbf{C}}} \begin{bmatrix} \dot{\mathbf{u}} \\ \dot{\mathbf{q}} \end{bmatrix} + \underbrace{\begin{bmatrix} \mathbf{K}_{3N\times3N} & \mathbf{0}_{3N\times N} \\ \mathbf{0}_{N\times3N} & \mathbf{k}_{N\times N} \end{bmatrix}}_{\bar{\mathbf{K}}} \begin{bmatrix} \mathbf{u} \\ \mathbf{q} \end{bmatrix} = \begin{bmatrix} \mathbf{Q} \\ \mathbf{f} \end{bmatrix}. \tag{S40}$$

To solve Eq. (S40) using commercially available code `ode45` in MATLAB, we rewrite Eq. (S40) into a set of first-order differential equations as

$$\mathbf{A}\dot{\mathbf{x}} + \mathbf{B}\mathbf{x} = \mathbf{y}, \tag{S41}$$

where

$$\mathbf{A} = \begin{bmatrix} \bar{\mathbf{C}}_{4N\times4N} & \bar{\mathbf{M}}_{4N\times4N} \\ \bar{\mathbf{M}}_{4N\times4N} & \mathbf{0}_{4N\times4N} \end{bmatrix}, \quad \mathbf{B} = \begin{bmatrix} \bar{\mathbf{K}}_{4N\times4N} & \mathbf{0}_{4N\times4N} \\ \mathbf{0}_{4N\times4N} & -\bar{\mathbf{M}}_{4N\times4N} \end{bmatrix}, \quad \mathbf{x} = \begin{bmatrix} \mathbf{u}_{3N\times1} \\ \mathbf{q}_{N\times1} \\ \dot{\mathbf{u}}_{3N\times1} \\ \dot{\mathbf{q}}_{N\times1} \end{bmatrix}, \quad \mathbf{y} = \begin{bmatrix} \mathbf{Q}_{3N\times1} \\ \mathbf{f}_{N\times1} \\ \mathbf{0}_{3N\times1} \\ \mathbf{0}_{N\times1} \end{bmatrix}. \tag{S42}$$

Finally, the collective self-oscillation of the active wire can be studied using the discrete model based on Eq. (S41). Fig. S7b shows the phase portrait of a particle in $x = 230$ mm of the active wire, and Fig. S7c shows the displacement profiles of the active wire at different time steps attained using both the continuum model performed in COMSOL Multiphysics (solid lines) and the discrete model built in MATLAB (solid dots). In the calculations, we consider an active wire with 15 unit cells with $l_w = 450$ mm, $D = 0.15$ N $\cdot$ s/m^2, $\omega_c = 700$ rad/s, $\mu = 0.5$, $R = 1 \times 10^3$ A/V, $V_{th} = 1$ V. Clearly, excellent agreement between the continuum and discrete models can be seen in both figures. It is thus safe to conclude that the time-dependent nonlinear numerical simulations performed in the manuscript are reliable.

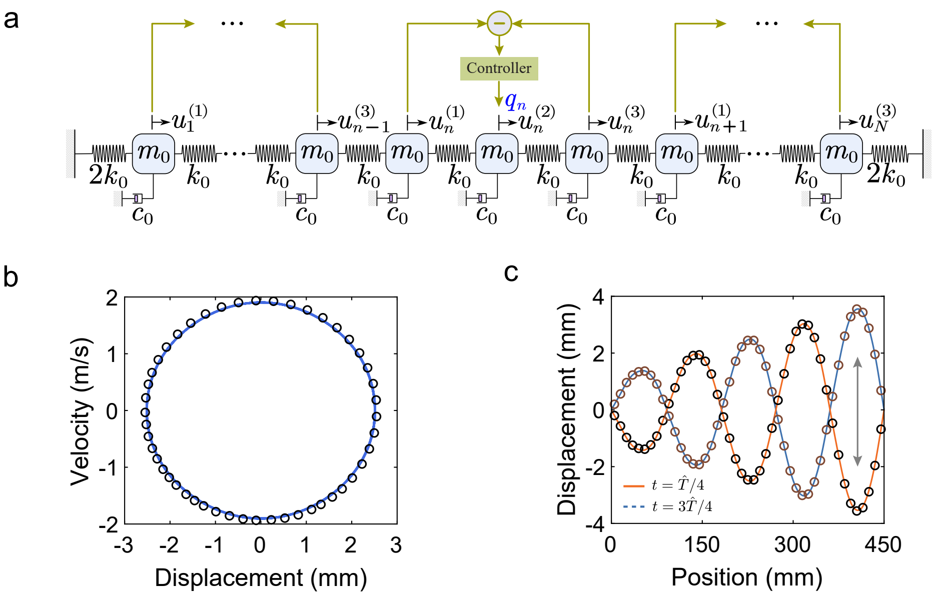

Fig. S7: (a) Schematic of the discrete mass-spring model of the active wire consisting of N unit cells. (b) Phase portrait of a particle in $x = 230$ mm of the active wire. (c) Displacement profiles of the active wire at different time steps. In (b) and (c), we show the results from both the continuum model performed in COMSOL Multiphysics (solid lines) and the discrete model built in MATLAB (solid dots).

S11. LINEARIZATION OF THE DISCRETE MODEL

Using the discrete model developed in the previous section, it is interesting to calculate the eigenfrequencies of the finite active mass-spring chain and compare them with the eigenfrequencies obtained from the continuum model in the main text. For this purpose, we first rewrite Eq. (S40) by ignoring the nonlinear terms as

$$\underbrace{\begin{bmatrix} \mathbf{M}_{3N\times 3N} & \mathbf{0}_{3N\times N} \\ \mathbf{0}_{N\times 3N} & \mathbf{m}_{N\times N} \end{bmatrix}}_{\tilde{\mathbf{M}}} \begin{bmatrix} \ddot{\mathbf{u}} \\ \ddot{\mathbf{q}} \end{bmatrix} + \underbrace{\begin{bmatrix} \mathbf{C}_{3N\times 3N} & \mathbf{0}_{3N\times N} \\ -\boldsymbol{\alpha}_{N\times 3N} & \mathbf{c}_{N\times N} \end{bmatrix}}_{\tilde{\mathbf{C}}} \begin{bmatrix} \dot{\mathbf{u}} \\ \dot{\mathbf{q}} \end{bmatrix} + \underbrace{\begin{bmatrix} \mathbf{K}_{3N\times 3N} & -\boldsymbol{\beta}_{3N\times N} \\ \mathbf{0}_{N\times 3N} & \mathbf{k}_{N\times N} \end{bmatrix}}_{\tilde{\mathbf{K}}} \begin{bmatrix} \mathbf{u} \\ \mathbf{q} \end{bmatrix} = \begin{bmatrix} \mathbf{0} \\ \mathbf{0} \end{bmatrix}, \tag{S43}$$

where the entities of matrices $\boldsymbol{\alpha}_{N\times 3N}$ and $\boldsymbol{\beta}_{3N\times N}$ are $\alpha_{n,3n-2} = R_q$, $\alpha_{n,3n} = -R_q$, $\beta_{3n-1,n} = 1$, and all the other entities are zero. We assume $[\mathbf{u}, \mathbf{q}]^T = \hat{\mathbf{X}} e^{i\omega t}$, and substitute it into Eq. (S43). A quadratic eigenvalue problem can then be formed as

$$(-\omega^2 \tilde{\mathbf{M}} + i\omega \tilde{\mathbf{C}} + \tilde{\mathbf{K}})\hat{\mathbf{X}} = \mathbf{0}. \tag{S44}$$

By calculating the eigenfrequencies ω in Eq. (S44), we can determine whether the active wire stays unstable to induce biased self-oscillation. Again, we compute those eigenfrequencies of an active wire and plot them in Fig. S8 in comparison with those from the continuum model performed in COMSOL Multiphysics. In the figure, $l_w = 450$ mm, $D = 0.15$ $\mathrm{N \cdot s/m^2}$, $\omega_c = 700$ rad/s, $\mu = 0.5$, $V_{th} = 1$ V. As expected, excellent agreement between the FE and discrete models is observed in the figure.

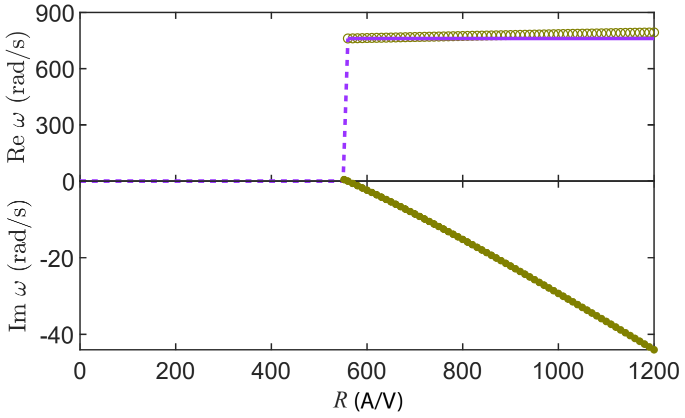

Fig. S8: Eigenfrequencies of an active wire with different amplification ratios R. The circles are the results from the discrete model; the dashed and solid curves are attained from the continuum model performed in COMSOL Multiphysics.

S12. EFFECTS OF THE WIRE DIAMETER ON SELF-OSCILLATIONS

In general, the wire's diameter d directly affects the mass per unit length in a string model that ignores bending stiffness, i.e. $\rho = \rho_0 \pi d^2/4$, where ρ_0 is the mass per unit volume. Using parameters $\rho_0 = 8.96$ g/cm^3, $T = 2$ N, $D = 0.15$ N $\cdot$ s/m^2, $\eta = 0$, $B = 1$ T, $\omega_c = 700$ rad/s, $\mu = 0.5$, $l = 10$ mm, $R = 1000$ A/V, and the total length of the active wire $l_w = 0.45$ m, Fig. S9 shows the effects of the wire diameter d on self-oscillation. Given that the string model neglects the bending stiffness, we limit our discussion to a reasonable range of variation, namely the diameter increases from 0.1 mm to 0.9 mm, and the mass per unit length $\rho = \rho_0 \pi d^2/4$ varies from 4.5 g/m to 5.7 g/m. Within this range, the oscillation frequency exhibits a periodic pattern of peaks and troughs. This behavior is accompanied by the emergence of six distinct modes of oscillation, each associated with specific diameter ranges and visually represented by varying mode shapes. Again, the ability to manipulate both the frequency and mode shape independently through adjustments in wire diameter confirms the versatility of our active wire design.

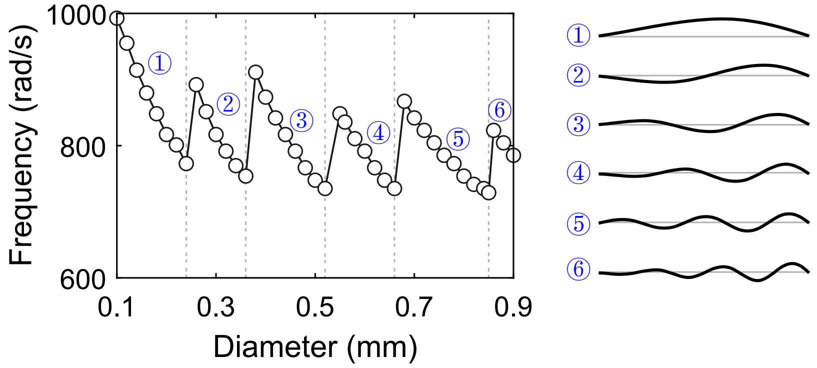

Fig. S9: Effects of the wire diameter on self-oscillations.

S13. POTENTIAL EXPERIMENTAL REALIZATION

The experimental demonstration of self-oscillation of the active wires would involve a range of well-planned procedures from wire and circuit fabrication and setup to signal and vibration tests and measurements. We elaborate on those procedures below and highlight potential challenges and possible strategies based on our current understanding.

1. **Wire Design and Fabrication.** For experimental realization, thin copper wire can be a suitable choice thanks to its favorable electrical and mechanical properties: (i) Electrically, it has relatively high conductivity and low resistance, leading to high energy efficiency in active wire designs; (ii) Mechanically, it is extremely flexible when bending but can support sufficient load under tension, making transverse motion much easier, which, at the

same time, can be controlled by the tensile force. To form the active wire, segments of the copper wire should be mechanically connected to maintain flexibility in bending and electrically isolated to separate sensors from actuators. For this purpose, tiny connection parts should be delicately designed and fabricated by 3D printing.

2. **Circuit Design and Fabrication.** All the circuits in the active wire, including a voltage amplifier, a nonlinear saturation circuit, a second-order low-pass filter, and a current amplifier, are standard components available in the market. However, a few points need to be carefully considered when designing and fabricating those circuits. (i) Signals from the sensing wire could be relatively weak. Voltage amplifiers used in electroencephalography and electromyography could be better choices due to their high sensitivity. (ii) A relatively high current would be demanded to produce visible oscillations. Power management chips could be potential solutions to supply the necessary current. (iii) Electromagnetic feedback in actuators should be carefully considered. Again, this can be addressed using power management chips. (iv) Electromagnetic interference between sensing and actuating should be minimized, where electromagnetic shielding cables may need to be implemented.

3. **Testing and Measurement Procedures.** To begin the experimental tests, a unit cell of the active wire can be tested and measured first. The magnetic field can be produced by sandwiching the wires between two permanent magnets. Sensing wires and their voltage amplifiers can be tested by using a shaker to excite the wire, measuring the velocity of the wire with a laser vibrometer, and acquiring sensing signals using an oscilloscope. The actuating wire and its current amplifier can be examined by inputting a signal into the amplifier and measuring the vibrations of the wire using a laser vibrometer. Other electrical circuits can be tested individually by connecting them to a function generator and an oscilloscope. After that, all the electrical circuits can connect to the copper wires, and the mechanical responses of the unit cell can be tested and measured. After the successful testing, the unit cells can be duplicated to form a 1D active wire, and its self-oscillation can be measured using a laser vibrometer.

Additionally, since our active wire shares similar principles as that in electromagnetic motors, it can be expected that the energy loss due to Joule heating should not be too large and is manageable. To mitigate energy losses, material selection offers a critical avenue. Employing high-conductivity materials, such as copper used in this study, for wire segments is able to reduce resistive losses. A complementary strategy involves preventing structural degradation or overheating. For instance, the saturation circuit design (Equation 2, Figure 1c) inherently limits Joule heating by imposing a saturation threshold on the applied voltage. Additionally, optimizing thermal management in specific operating environments, such as aqueous settings, can enhance heat dissipation. Submerging components in water, for example, leverages the medium's high thermal conductivity to minimize localized temperature rise and stabilize system performance. Overall, by integrating these strategies, the impact of the Joule effect could be significantly reduced, leading to more efficient and reliable applications of our active wires.

---

[1] K. F. Graff, *Wave motion in elastic solids* (Courier Corporation, 2012).
